# Free-Volume Nanostructurization in Ga-Modified As_2_Se_3_ Glass

**DOI:** 10.1186/s11671-016-1237-8

**Published:** 2016-01-13

**Authors:** Ya. Shpotyuk, A. Ingram, O. Shpotyuk, A. Dziedzic, C. Boussard-Pledel, B. Bureau

**Affiliations:** Department of Electronics, Ivan Franko National University of Lviv, 107, Tarnavskogo str., 79017 Lviv, Ukraine; Centre for Innovation and Transfer of Natural Sciences and Engineering Knowledge, University of Rzeszow, 1, Pigonia str., 35-959 Rzeszow, Poland; Laboratoire Verres et Céramiques, UMR-CNRS 6226, Université de Rennes 1, 35042 Rennes, Cedex France; Opole University of Technology, 75, Ozimska str., 45370 Opole, Poland; Vlokh Institute of Physical Optics, 23, Dragomanov str., 79005 Lviv, Ukraine; Institute of Physics, Jan Dlugosz University, 13/15, Armii Krajowej al., 42200 Czestochowa, Poland

**Keywords:** Chalcogenides, Nanostructurization, Phase separation, Crystallization, Positron annihilation lifetime spectroscopy

## Abstract

Different stages of intrinsic nanostructurization related to evolution of free-volume voids, including phase separation, crystalline nuclei precipitation, and growth, were studied in glassy As_2_Se_3_ doped with Ga up to 5 at. %, using complementary techniques of positron annihilation lifetime spectroscopy, X-ray powder diffraction, and scanning electron microscopy with energy-dispersive X-ray analysis. Positron lifetime spectra reconstructed in terms of a two-state trapping model testified in favor of a native void structure of g-As_2_Se_3_ modified by Ga additions. Under small Ga content (below 3 at. %), the positron trapping in glassy alloys was dominated by voids associated with bond-free solid angles of bridging As_2_Se_4/2_ units. This void agglomeration trend was changed on fragmentation with further Ga doping due to crystalline Ga_2_Se_3_ nuclei precipitation and growth, these changes being activated by employing free volume from just attached As-rich glassy matrix with higher content of As_2_Se_4/2_ clusters. Respectively, the positron trapping on free-volume voids related to pyramidal AsSe_3/2_ units (like in parent As_2_Se_3_ glass) was in obvious preference in such glassy crystalline alloys.

## Background

Ga-modified chalcogenide glasses (ChG) are known to be of high importance in view of their perspectives for modern IR photonics as active media with improved optical functionality, revealed, in part, when these glasses are doped with rare earth (RE) activators such as Pr^3+^, Dy^3+^, Tb^3+^, Er^3+^, and Nd^3+^ [1–9]. Such ChG demonstrates an obvious tendency to nanostructurization by forming intrinsic inhomogeneities because of strong Ga affinity to chemical interaction with chalcogens, this process being governed by Ga content and preferential type of its environment in parent glass matrix [4, 10–16]. In dependence on these pre-requisites, extra Ga additions can result in phase separation, nucleation and, finally, crystal growth, leading to stabilization of different crystalline Ga_2_Se_3_ polymorphs. Thus, under small Ga content (2–3 at. %) added in mixed Se–Te environment of TAS-235 glass (e.g., glassy g-As_30_Se_50_Te_20_ alloy), the nanoscale droplets of dominated γ-Ga_2_Se_3_ phase (a few hundreds of nanometers in sizes) can be displayed, while at more enhanced Ga content reaching 5–10 at. %, this process extends over a microscale, when these crystallites grow to a few micrometers in sizes [8, 13]. In contrast, in Se-rich environment of Ge-based GeSe_2_–Ga_2_Se_3_ glass at heat treatment not too far above *T*_*g*_, these Ga additions provoke formation of some multication crystallites like GeGa_4_Se_8_ [14–16] or Ga_2−δ_Ge_δ_Se_3_ [12]. Crystallite growth and stabilization in ChG matrices is accompanied by complicated changes stretching over both atomistic (atomic-specific) and void (atomic-deficient) structural levels. The latter is related to the evolution of some free-volume entities (typically sub-nanoscale voids, vacancies, vacancy-like clusters, etc.), when inner holes are agglomerated to form spaces of reduced electron density available for orientation stabilization of growing crystallites or, conversely, these holes are fragmented on smaller parts ensuring energetically favorable localization for growing crystallites in a predominantly glass environment [13]. In case of technologically controlled crystallization, it is possible to manufacture an important class of glass ceramics transparent in IR region, which possess much better mechanical reliability than their glassy counterparts [17]. But in most cases, these crystallization processes are undesirable, especially when ChG should be doped with RE ions to get tunable, high-power, secondary remote mid-IR sources [9], or drawn into fiber to produce active media for optical biosensing [18].

In this work, the physical peculiarities of Ga-affected nanostructurization associated with subsequent stages of glass structure modification (phase separation, nucleation, and crystallization), overall described at atomic-deficient void level, are comprehensively studied in g-As_2_Se_3_, one of the well-known canonical representatives of functional chalcogenide photonics [19].

## Methods

The studied samples of Ga_*x*_(As_0.4_Se_0.6_)_100−*x*_ (*x* = 0, 1, 2, 3, 4, 5) alloys were prepared from high-purity elemental constituents (5 N or more) by conventional melt-quenching technique as described elsewhere [9, 11]. Total weight of ingredients inserted in silica ampoules of 10 mm in diameter used for melting was 30 g. The ampoule was sealed under a vacuum and heated at 900 °C in a rocking furnace for 10 h, followed by quenching into room temperature water from 700 °C. Then, these alloys were annealed during 5 h at 10 K below glass transition to remove mechanical strains that appeared during quenching, cut into disks of ~2 mm in thickness, and finally, polished to high optical quality.

The crystalline state of the samples was controlled with X-ray powder diffraction (XRPD), experimental data being collected in the transmission mode on a STOE STADI P diffractometer (Cu Kα_1_-radiation). The crystal structures of phases were refined by the Rietveld method with the program FullProf.2k (v. 5.40) [20]. The surface morphology of fresh cut sections of the prepared alloys was tested using scanning electron microscope (SEM) with energy-dispersive spectroscopy (EDS) analyzer FEI QUANTA 3D 200i (Hillsboro, OR, USA).

Positron annihilation lifetime (PAL) spectra were registered using fast coincidence system ORTEC of 230 ps resolution (the full width at half maximum) operated at high-stabilized normal measuring conditions. The pair of identical plane-parallel samples of each composition in sandwich geometry was employed for the measurements. The source contribution from ^22^Na isotope of low activity was taken at the level of 12 % (*τ* = 0.372 ns). To ensure reliable PAL data, three independent measuring cycles (with near 1 M elementary positron annihilation events) were performed. Experimental results were fitted by two single exponents under normalized intensities (*I*_1_ + *I*_2_ = 1) using LT 9.0 program [21], the corresponding accuracies in lifetimes *τ*_*i*_ and intensities *I*_*i*_ being not worse ±0.005 ns and 0.5 %, respectively. Mathematical formalism of the known two-state positron trapping model with only one kind of traps [22–26] was utilized to parameterize mean *τ*_av_ and defect-free bulk *τ*_b_ lifetimes, as well as trapping rate in defects *κ*_d_. In addition, the difference between defect-related *τ*_d_ = *τ*_2_ and bulk positron lifetimes (*τ*_2_−*τ*_b_) was taken as a signature of size of positron traps in terms of equivalent number of vacancies, and *τ*_2_/*τ*_b_ ratio was ascribed to nature of these defects [22]. The fraction of trapped positrons *η* = *τ*_1_⋅*κ*_*d*_ was also controlled in this research.

## Results and Discussion

**Fig. 1:**
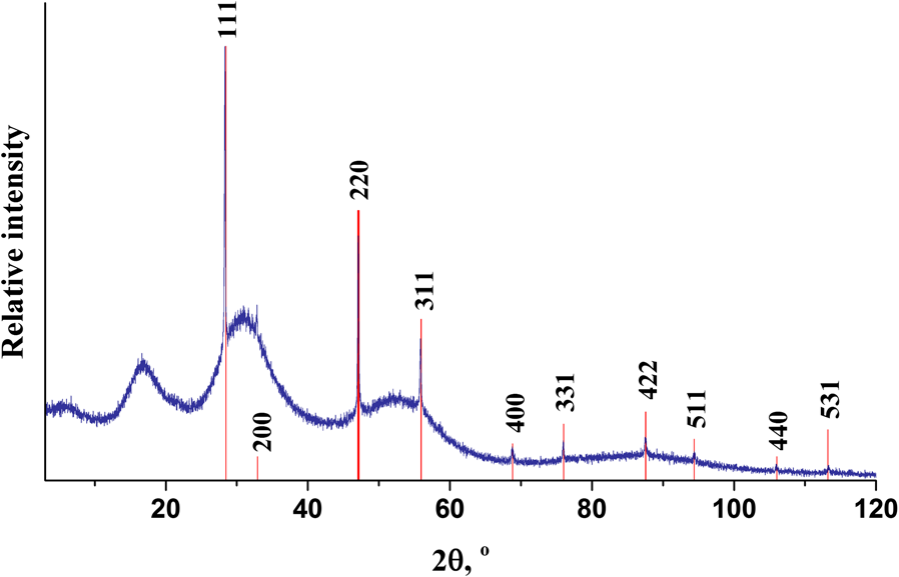
Experimental XRPD pattern of g/c-Ga_5_(As_0.4_Se_0.6_)_95_ sample (*blue color*) in comparison with theoretical diffraction pattern of Ga_2_Se_3_ high-temperature phase of ZnS structural type. (*red color*, *hkl* indexes are indicated above the peaks)

Different stages of intrinsic nanostructurization can be activated in g-As_2_Se_3_ in dependence on the amount of added Ga [9, 11]. Firstly, at small enough Ga content within studied Ga_*x*_(As_0.4_Se_0.6_)_100−*x*_ cut section not exceeding 3 at. %, the melt-quenched alloys are fully in a glassy state, since no any sharp reflexes (but only wide-stretched halos typical for amorphous substances) are detected on the XRPD patterns [9]. Further, at higher Ga content, some crystallites of low-temperature α-Ga_2_Se_3_ phase appear in glassy crystalline g/c-Ga_4_(As_0.4_Se_0.6_)_96_ alloy, the crystallized phase being well identified due to six sharp XRPD peaks corresponding to one of cubic Ga_2_Se_3_ polymorphs with a space group of $F\overline{4}3m$ (JCPDS ICDD card no. 89-7201) [9, 27–29]. The most pronounced crystallization effect is revealed in g/c-Ga_5_(As_0.4_Se_0.6_)_95_, where ten separate XRPD peaks centered near 28.5°, 33°, 47°, 56°, 69°, 76°, 87.5°, 94.5°, 106°, and 113° 2Θ are detected (Fig. 1). These peaks can be well indexed assuming *fcc* Ga_2_Se_3_ structure of zinc blende type ($F\overline{4}3m$ space group), which allow to estimate the unit cell lattice parameter *a* = 5.4576(6) Å (with respective unit cell volume *V* = 162.55(3) Å^3^). In accord with similar behavior in Ga-doped g-As_30_Se_50_Te_20_ [8, 13], this effect can be ascribed to preferential formation of high-temperature γ-Ga_2_Se_3_ phase.

**Fig. 2:**
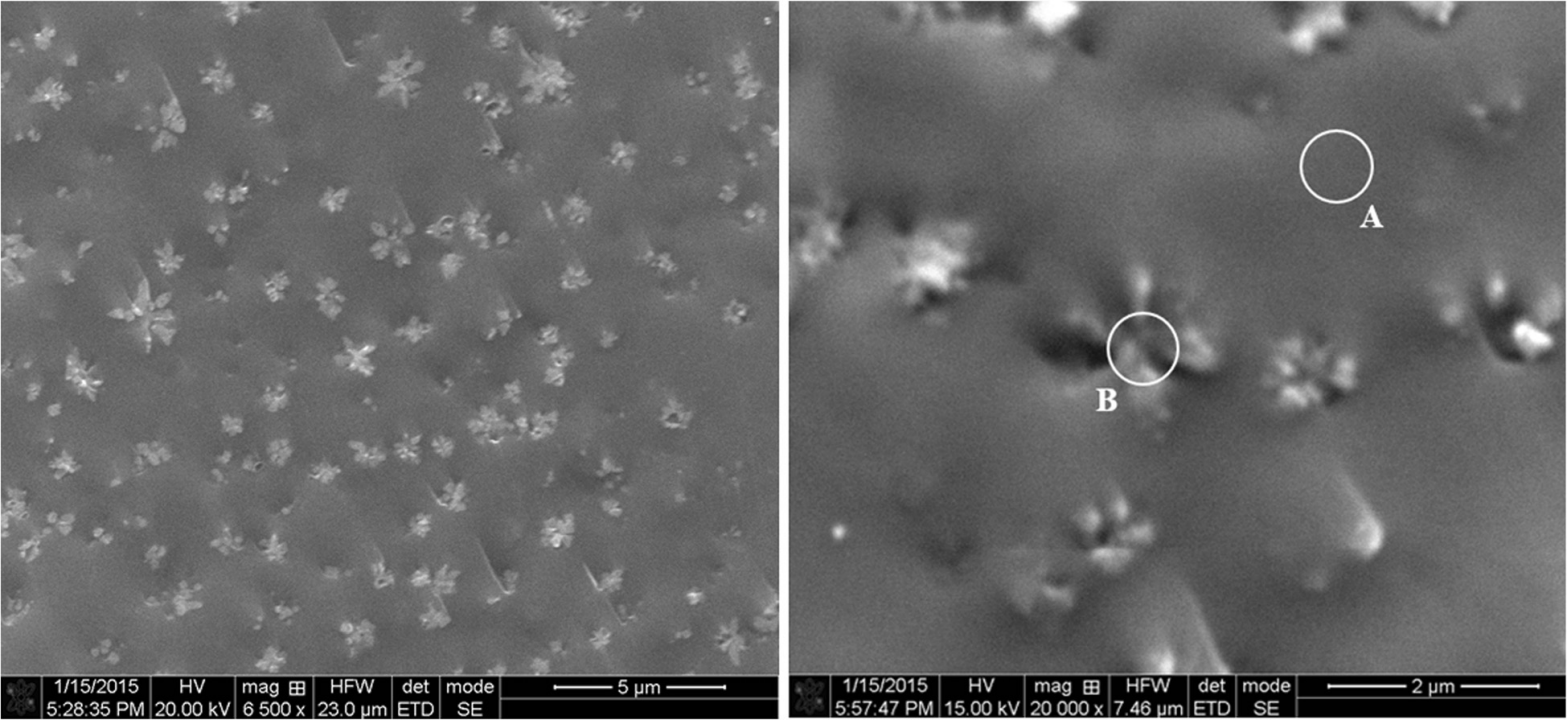
SEM micrograph of freshly prepared surface cut section of g/c-Ga_5_(As_0.4_Se_0.6_)_95_ alloy, showing regions without visible inclusions (*spot A*) and containing Ga_2_Se_3_ crystallites (*spot B*)

**Fig. 3:**
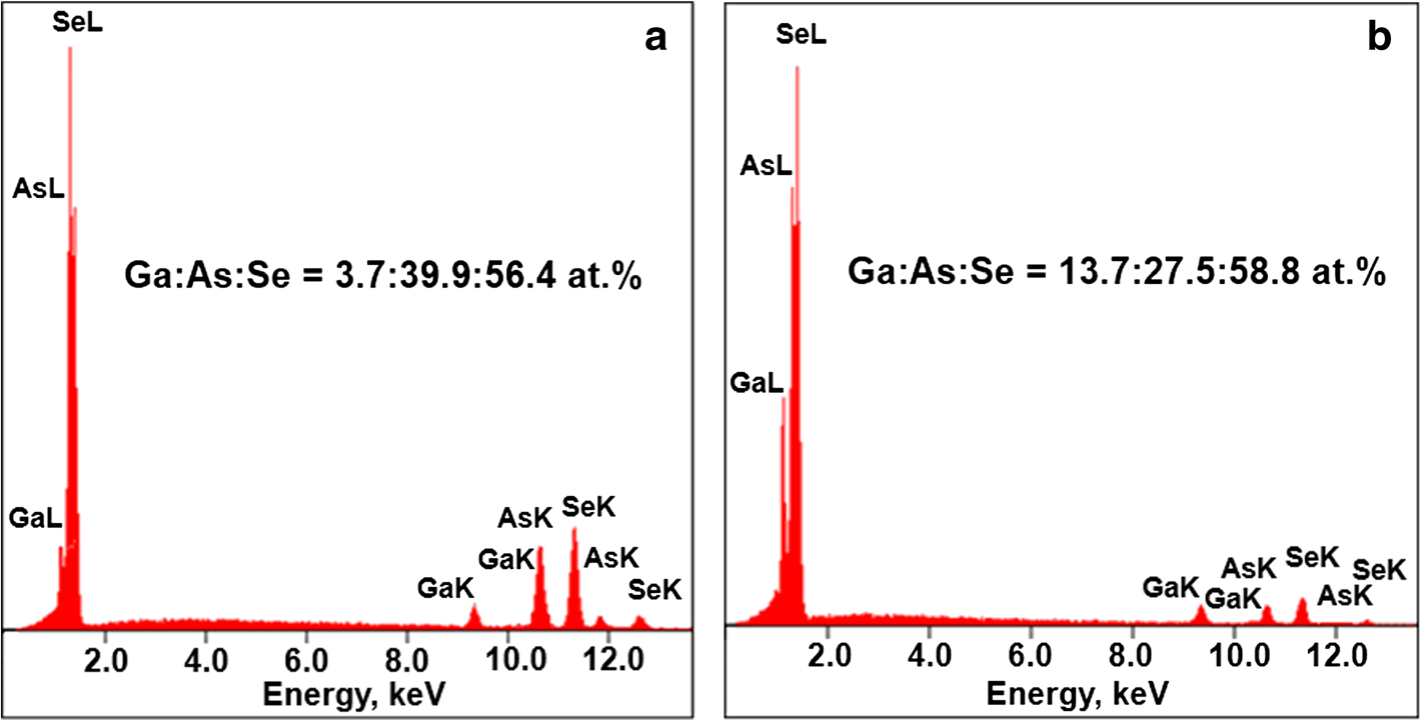
EDS spectra detected from A (**a**) and B (**b**) spots at the cut surface of g/c-Ga_5_(As_0.4_Se_0.6_)_95_ showing disproportionalities in chemical composition

Under this Ga content, the crystallization is most evident, the cones of four to six separate Ga_2_Se_3_ crystallites (each less than 1 μm) being well distinguished at cut surface of g/c-Ga_5_(As_0.4_Se_0.6_)_95_ alloys (Fig. 2). The chemical composition of the sample was checked in two different spots, these being uniform surface area without visible inclusions (A) and one with some agglomerated crystallites (B). The corresponding EDS spectra from these spots detected up to 15 keV (current 0.65 nA) are shown in Fig. 3. Only arsenic As, selenium Se, and gallium Ga were defined as main elements responsible for the observed peaks on these spectra, located respectively to their Lα and Kα states. Despite uncertainties due to large scatter in the experimental points, a sharp increase in Ga and decrease in As content was detected from a crystallite-covered area. In opposite, the uniform sample matrix near crystallites was enriched on As and deficient on Ga, while Se content was nearly the same over a whole sample’s surface. These results testify that extraction of Ga_2_Se_3_ phase is indeed the most plausible reason for growing crystallites.

**Fig. 4:**
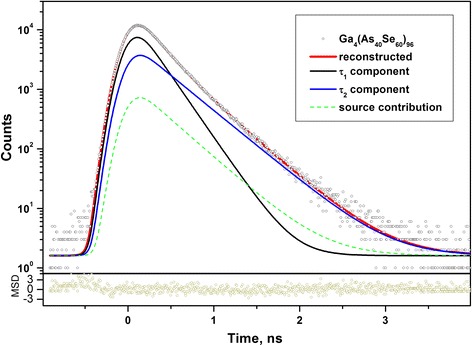
Raw PAL spectrum of crystallized Ga_4_(As_0.4_Se_0.6_)_96_ alloy reconstructed from a two-component fitting at the general background of source contribution (*bottom inset* shows statistical scatter of variance)

The above processes essentially modify atomic-deficient void structure of Ga_*x*_(As_0.4_Se_0.6_)_100−*x*_ alloys, as it follows from PAL spectra reconstructed within the two-component fitting procedure (Fig. 4) and best-fit positron trapping parameters gathered in Table 1. Since neither average *τ*_av_ nor defect-free bulk *τ*_b_ positron lifetimes are changed under Ga additions, the two-state trapping model based on one preferential type of positron traps [22–26] can be validated as most relevant to the physically realistic channel of positron annihilation in these alloys. The volumes of these traps (e.g., sizes of low-electron density spaces) in g or g/c alloys are reflected by defect-related *τ*_2_ lifetimes, while their contents being proportional to intensities of the second fitting components (*I*_2_) [22]. Thus, the positron trapping modes correspondingly recalculated in respect to the above fitting parameters, including trapping rate in defects *κ*_d_, (*τ*_2_−*τ*_b_) difference, *τ*_2_/*τ*_b_ ratio, and fraction of trapped positrons *η*, can be ascribed to the same type of traps as those characteristic for g-As_2_Se_3_ [23, 30–33], which are subjected to Ga-activated compositional modification within the Ga_*x*_(As_0.4_Se_0.6_)_100−*x*_ system.

**Table 1:** Fitting parameters and positron trapping modes describing two-component reconstructed PAL spectra of Ga_*x*_(As_0.4_Se_0.6_)_100−*x*_ alloys

Sample, state	Fitting parameters			Positron trapping modes					
	*τ*_1_	*τ*_2_	*I*_2_	*τ*_av_	*τ*_b_	*κ*_d_	*τ*_2_ *−τ*_b_	*τ*_2_ */τ*_b_	*η*
	ns	ns	a.u.	ns	ns	ns^−1^	ns	–	–
g-As_2_Se_3_	0.210	0.360	0.462	0.279	0.260	0.92	0.10	1.39	0.19
g-Ga_1_(As_0.4_Se_0.6_)_99_	0.216	0.371	0.408	0.279	0.261	0.78	0.11	1.42	0.17
g-Ga_2_(As_0.4_Se_0.6_)_98_	0.223	0.382	0.401	0.287	0.267	0.75	0.11	1.43	0.17
g-Ga_3_(As_0.4_Se_0.6_)_97_	0.211	0.365	0.457	0.281	0.261	0.91	0.10	1.39	0.19
g/c-Ga_4_(As_0.4_Se_0.6_)_96_	0.204	0.359	0.488	0.280	0.258	1.03	0.10	1.39	0.21
g/c-Ga_5_(As_0.4_Se_0.6_)_95_	0.207	0.362	0.462	0.279	0.258	0.95	0.10	1.40	0.20

In g-As_2_Se_3_, the positron trapping is defined by spatial distribution of low-electron density spaces (native free-volume voids) formed within *cycle-type* formations of chalcogen-interlinked polyhedrons, such as AsSe_3/2_ pyramids (the fractional subscript means atoms simultaneously belonging to two neighboring units) [23, 31]. Despite deviation in the number of elements forming such cycle-type formations in a glass, they can be considered as network remainders of strict 12-membered cycles in a crystalline structure of rhombohedral As_2_Se_3_ [34]. In void structure evolution in ChG, an essential role belongs to spaces in the nearest atomic surrounding, which are free of electron density due to directionality of covalent bonds, e.g., bond-free solid angles (BFSA) in terms of Kastner [35]. Depending on electronegativity of neighbors [36], these intrinsic local spaces can be associated with effective neutral (Se atoms in –Se–**Se**–Se– chain- and ring-like fragments), positive (As atoms in the top of AsSe_3/2_ pyramids), or negative (Se atoms within heteronuclear =As–**Se**–As= bridges, e.g., in the bottom of neighboring AsSe_3/2_ pyramids) electrical charge [23, 31, 37]. So the most efficient positron traps in a covalent-bonded network of g-As_2_Se_3_ represent geometrical free-volume spaces within corresponding cycle-type formations of interlinked AsSe_3/2_ pyramids (atomic-accessible void core) surrounded by low-electron density spaces originated from atomic environment due to strict directionality of covalent chemical bonds (atomic-inaccessible void shell). The possible configurations of some void shells (e.g., BFSA) in ChG systems are considered in more detail elsewhere [23, 37, 38]. If only geometrical free volumes (void cores) have been decisive in a positron trapping in ChG, then defect-related positron lifetime *τ*_2_ would be in strong correlation with molar volume in a g-As–Se system. However, an obviously opposite tendency is observed in g-As–Se, e.g., increase in *τ*_2_ positron lifetime with going from looser Se-rich towards stoichiometric As_2_Se_3_ and As-rich glasses [31], thus meaning an importance of contribution from agglomerated BFSA (void shells) surrounding geometrical voids. From a standpoint of positron trapping [22], the heteroatomic environment around a chalcogen atom has an obvious preference. Indeed, because of difference in the electronegativity [36], such low-electron density sites carry an effective negative charge, thus transforming corresponding free-volume void in a prototype of cation vacancy in a crystal. This local electrical charge distribution is not characteristic of pure homoatomic Se chains or rings, resulting in reduced positron trapping in Se-rich ChG [31, 32].

**Fig. 5:**
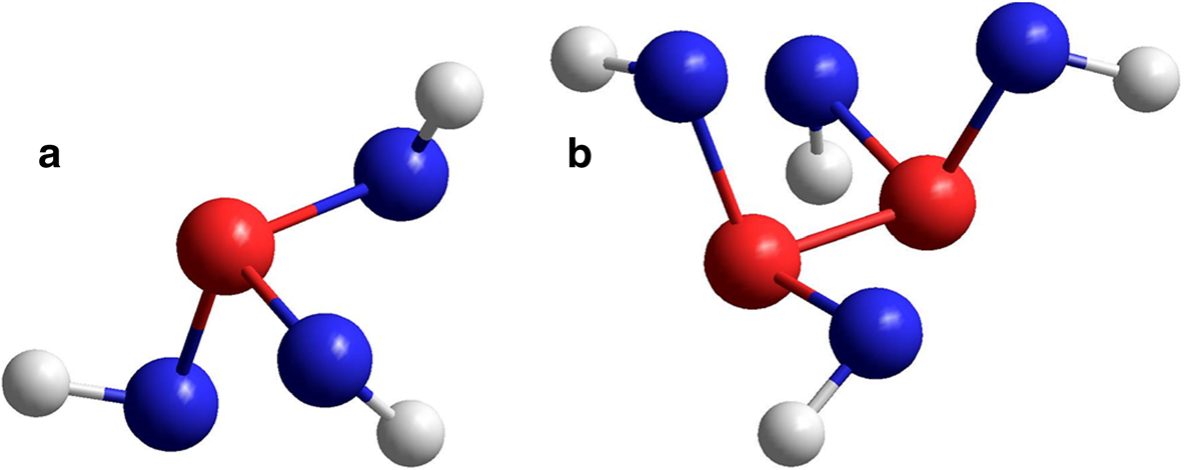
Geometrically optimized configurations of some atomic clusters in g-As-Se computed by ab initio quantum chemical modeling with RHF/6-311G^*^ basis set [39]. **a** AsSe_3/2_ pyramid (two of three Se atoms contribute with their BFSA to one hemisphere). **b** As_2_Se_4/2_ bridge (all four Se atoms contribute with their BFSA to one hemisphere)

**Fig. 6:**
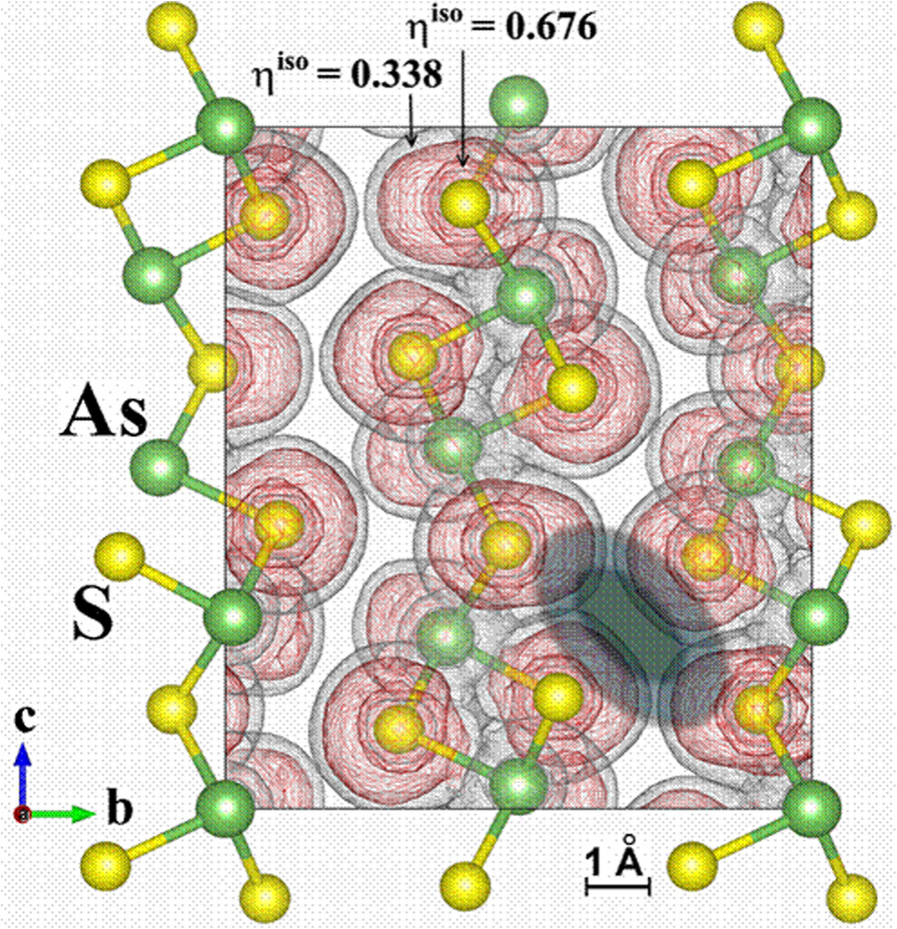
Electron density distribution in mineral As_2_S_3_ orpiment visualized with Exciting package [40]: unit cell is along [100] with two isosurface levels of electron localization function (ELF = 0.676, 0.338); the boundaries of free-volume void evolving core and surrounding shell are dark-distinguished

In the network of Se-interlinked AsSe_3/2_ pyramids forming the structure of g-As_2_Se_3_, two of three Se atoms in the bottom of the AsSe_3/2_ pyramid contribute with their BFSA towards free-volume void, while the third Se atom contradicts this because of electron density cone of a covalent bond directed in an opposite hemisphere as void. The geometrically optimized configuration of such AsSe_3/2_ pyramidal cluster computed with ab initio quantum chemical modeling [39] is shown in Fig. 5. Thus, the most expected positron traps in g-As_2_Se_3_ can be imagined as free-volume voids surrounded by neighboring BFSA originated from two Se atoms (in heteroatomic =As–**Se**–As= environment) of the AsSe_3/2_ pyramid. The expected volumes of such positron traps (near ~90 Å^3^) can be simply estimated from corresponding defect-related positron lifetime *τ*_2_ ≅ 0.36 ns in respect to Jensen’s calculations [33]. Possible configuration of such void in orpiment As_2_S_3_ crystal, the known crystalline counterpart of As_2_Se_3_ [34], reproduced in respect to electronic structure calculations for mineral arsenic sulfide As_2_S_3_ performed using Exciting package [40] is depicted in Fig. 6. Undoubtedly, such voids remain in melt-quenched state, thus defining preferential positron trapping in ChG, while voids surrounded by As-based BFSA are rather ineffective for trapping because of repulsive potential for positrons [31, 32]. With transition to over-stoichiometric As-rich ChG, the bridging As_2_Se_4/2_ units based on homopolar As–As bonds appear in a network of AsSe_3/2_ pyramids. Both pairs of Se atoms at the edges of the As–As bond contribute fully to free-volume void, since their BFSA are directed to one hemisphere without any restrictions on opposite directionality of Se atoms like in AsSe_3/2_ units (see Fig. 5). So the volumes of such voids grow (in full harmony with decreased atomic densities [34]), thus resulting in enhanced defect-related *τ*_2_ lifetimes for ChG consisting of these structural units, while their concentration (reflected in *I*_2_ intensity) essentially drops in view of chemical formulation [23, 41].

This effect is dominated in g-As_2_Se_3_ at small Ga addition, specifically in g-Ga_1_(As_0.4_Se_0.6_)_99_ and g-Ga_2_(As_0.4_Se_0.6_)_98_, where increase in *τ*_2_ lifetime from 0.360 to 0.382 ns (Table 1) is caused by transition to positron trapping on free-volume voids neighboring with Se-type BFSA of As_2_Se_4/2_ units. The bridging As_2_Se_4/2_ clusters appear due to deficit in Se environment for As atoms because of Ga doping at small concentration. Indeed, chemical interaction in a Ga–As–Se system is governed by Ga bonding. By accepting energies for homonuclear Ga–Ga, As–As, and Se–Se bonds as 34.1, 32.1, and 44.0 kcal/mol, respectively [36, 42], the energies for heteronuclear Ga–Se, Ga–As, and As–Se bonds can be estimated in respect to known Pauling’s formula [36] as 55.2, 37.2, and 41.7 kcal/mol, respectively. Thereby, the Ga dopants form polyhedra with Se atoms inserted in remainder of the As–Se network [8–11, 43, 44]. Because of higher atomic packing of Ga-doped ChG [8–11], this trend is accompanied by reduced second-component *I*_2_ intensity, trapping rate in defects *κ*_d_, and fraction of trapped positrons *η* in addition to enlarged *τ*_2_ lifetime, (*τ*_2_−*τ*_b_) difference, and *τ*_2_/*τ*_b_ ratio (Table 1).

Another g-Ga_3_(As_0.4_Se_0.6_)_97_ alloy, possessing positron trapping modes which are rather close to the ones of g-As_2_Se_3_ (Table 1), is obviously exceptional from the above correlation line. Defect-related annihilation channel in this ChG is presumably distorted due to crystalline nuclei precipitation of separated Ga_2_Se_3_ phase, like it occurred in 80GeSe_2_–20Ga_2_Se_3_ glass under thermally induced “cold” crystallization [16]. At this stage, the Ga_2_Se_3_ crystallites do not grow enough to be detected quite reliably with XRPD. The nuclei precipitation process is activated by employing free volume of just attached As-rich matrix with preferential content of bridging As_2_Se_4/2_ clusters. Correspondingly, the defect-related *τ*_2_ lifetime drops down to the value of ~0.36 ns, which is characteristic of positron trapping on free-volume voids within AsSe_3/2_-based cycle formations in stoichiometric g-As_2_Se_3_ (see Table 1).

This process is continued in g/c-Ga_4_(As_0.4_Se_0.6_)_96_ and g/c-Ga_5_(As_0.4_Se_0.6_)_95_ alloys due to further growth and precipitation of Ga_2_Se_3_ crystallites, which became XRPD-detectable at these conditions (Fig. 1). These crystallites are restricted in their growing in Se-deficient environment, thus being stabilized in the ChG by utilizing atomic-accessible free volumes of their nearest environment rich in As_2_Se_4/2_ clusters. In such alloys, the positron trapping on free-volume entities built of pyramidal AsSe_3/2_ units has an obvious preference, tending the overall annihilation-like void fragmentation without notable changes in sizes of positron traps (*τ*_2_ = ~0.36 ns) and enhanced second-component intensities *I*_2_, in full respect to the experimental data in Table 1.

## Conclusions

The nanostructurization processes related to evolution of free-volume voids were studied in glassy As_2_Se_3_ affected by different amounts of Ga additions (up to 5 at. %), using positron annihilation lifetime spectroscopy, the lifetime spectra being analyzed within a canonical two-component model with one preferential type of positron traps. It was shown that below 3 at. % of Ga, when glass formation was not essentially violated, the positron trapping is dominated on intrinsic voids associated with bond-free solid angles of bridging As_2_Se_4/2_ units, thus producing an increase in defect-related lifetime from 0.360 to 0.382 ns at the cost of reduced second-component intensity and positron trapping rate in defects. This void agglomeration trend was changed on opposite (void fragmentation) at greater Ga content in As_2_Se_3_ glass due to crystalline Ga_2_Se_3_ nuclei precipitation and growth. The observed crystallization processes were activated by employing free volume of just attached As-rich matrix with higher content of As_2_Se_4/2_ clusters. In such glassy crystalline alloys, the positron trapping on free-volume entities related to pyramidal AsSe_3/2_ units (as in parent As_2_Se_3_ glass) was in obvious preference, tending overall annihilation-like void fragmentation without notable changes in sizes of positron traps.

## Abbreviations

BFSA: bond-free solid angle
ChG: chalcogenide glass
EDS: energy-dispersive spectroscopy
PAL: positron annihilation lifetime
RE: rare earth
SEM: scanning electron microscope
XRPD: X-ray powder diffraction
